# Checking whether there is an increased risk of post-transplant lymphoproliferative disorder and other cancers with specific modern immunosuppression regimens in renal transplantation: Protocol for a network meta-analysis of randomized and observational studies

**DOI:** 10.1186/2046-4053-3-16

**Published:** 2014-02-22

**Authors:** Brian Hutton, Lawrence Joseph, Fatemeh Yazdi, Jennifer Tetzlaff, Mona Hersi, Madzouka Kokolo, Nicolas Fergusson, Alexandria Bennett, Chieny Buenaventura, Dean Fergusson, Andrea Tricco, Sharon Strauss, David Moher, Greg Knoll

**Affiliations:** 1 Ottawa Hospital Research Institute, Center for Practice Changing Research, 501 Smyth Road, K1H 8 L6 Ottawa, ON, Canada; 2 Department of Epidemiology and Community Medicine, University of Ottawa, Ottawa, ON, Canada; 3 McGill University Department of Epidemiology and Biostatistics, Montréal, QC, Canada; 4 Li Ka Shing Knowledge Institute, St Michael’s Hospital, Toronto, ON, Canada

**Keywords:** Renal transplant, malignancy, post-transplant lymphoproliferative disorder, systematic review, network meta-analysis

## Abstract

**Background:**

Patients undergoing renal transplant procedures require multi-agent immunosuppressive regimens both short term (induction phase) and long term (maintenance phase) to minimize the risk of organ rejection. There are several drug classes and agents for immunosuppression. Use of these agents may increase the risk of different harms including not only infections, but also malignancies including post-transplant lymphoproliferative disorder. There is a need to identify which regimens minimize the risk of such outcomes. The objective of this systematic review and network meta-analysis of randomized and observational studies is to explore whether certain modern regimens of immunosuppression used to prevent organ rejection in renal transplant patients are associated with an increased risk of post-transplant lymphoproliferative disorder and other malignancies.

**Methods/design:**

‘Modern’ regimens were defined to be those evaluated in controlled studies beginning in 1990 or later. An electronic literature search of Medline, Embase and the Cochrane Central Register of Controlled Trials has been designed by an experienced information specialist and peer reviewed by a second information specialist. Study selection and data collection will be performed by two reviewers. The outcomes of interest will include post-transplant lymphoproliferative disorder and other incident forms of malignancy occurring in adult renal transplant patients. Network meta-analyses of data from randomized and observational studies will be performed where judged appropriate based on a review of the clinical and methodological features of included studies. A sequential approach to meta-analysis will be used to combine data from different designs.

**Discussion:**

Our systematic review will include both single-agent and multi-agent modern pharmacotherapy regimens in patients undergoing renal transplantation. It will synthesize malignancy outcomes. Our work will also add to the development of methods for network meta-analysis across study designs to assess treatment safety.

**Trial registration:**

PROSPERO Registration Number: CRD42013006951

## Background

Kidney transplantation is the treatment of choice for patients with end-stage kidney disease [1,2]. Patients undergoing a renal transplant must be treated with immunosuppressive agents to prevent organ rejection. These medications have different mechanisms of action [3], but in some way disrupt the interaction and/or stimulation of the antigen-presenting cells or T-lymphocytes of the human immune system. Immunosuppression for renal transplantation includes both an *induction* phase and a *maintenance* phase [3]. Induction involves the use of one of many medications such as an anti-IL2 receptor antagonist (basiliximab or daclizumab), a polyclonal anti-T-cell antibody (e.g. anti-thymocyte globulin) or a monoclonal anti-T-cell antibody (e.g. anti-CD52, alemtuzumab, anti-CD3 or OKT3). The maintenance phase often involves two or three medications in combination, but may also be a single agent. Possible agents include calcineurin inhibitors (cyclosporin or tacrolimus), anti-metabolite agents (azathioprine, mycophenolate mofetil or mycophenolate sodium), steroids (prednisone), co-stimulation blocker (belatacept) and/or mammalian target of rapamycin inhibitors (sirolimus or everolimus) [4].

Immunosuppressive agents are associated with undesired outcomes, including infection and malignancy [5-9]. Of particular concern is a specific transplant-related cancer referred to as *post-transplant lymphoproliferative disorder* (PTLD), which has a one-year mortality rate as high as 40% [10]. A recent editorial noted a clear need to determine which immunosuppressant combinations minimize the occurrence of PTLD, as the types of drugs used for the induction and maintenance phases play a role in the rate and types of cancers seen [10-12]. From 2001 to 2010, 10,795 kidney transplants were performed in Canada, and there are currently 16,164 Canadians alive with a functioning renal transplant taking immunosuppressive agents [13]. Patients receiving immunosuppressive drugs following renal transplant are at an increased risk of cancers that shorten survival, reduce quality of life and have high treatment costs. It is important to determine if there are specific regimens that can minimize the risk of PTLD and other cancers.

Network meta-analysis [14-16] is a statistical approach used to compare different treatment regimens when there is both direct and indirect information. Our systematic review and network meta-analyses will compare the rates of PTLD and other cancers across the available regimens.

## Methods/design

### Research question and selection criteria

Our systematic review will address the following research question: In adults undergoing renal transplantation, is there evidence of an association between certain immunosuppressive regimens used in the induction and maintenance phases and the risk of PTLD and other cancers? The following criteria, summarized in the Population – Interventions/Comparators – Outcomes – Study design (PICOS) format, summarize the planned selection criteria for this review.

#### Population

The review will include data from both male and female adult patients who underwent a renal transplant. Patients who underwent either a first-time or repeat transplantation are eligible for inclusion.

### Intervention and comparators

The pharmacologic agents of relevance to this review are summarized in Table 1. It is anticipated that the majority of studies will involve combinations of these agents; however, monotherapy studies will also be included. Studies of steroid avoidance and withdrawal will also be included. It is possible that we will encounter comparisons involving the same pharmacologic agents but at varied doses across studies. Should this occur, clinical experts will be consulted to determine the extent to which such variations warrant ‘lumping’ into the same treatment node within our network. In addition to agent/combination level analyses, broader analyses of regimens such as regimens with ‘induction vs no induction’ and ‘depleting vs non-depleting’ may also be explored if feasible, as well as analyses related to the intensity of immunosuppression (related to both dose and duration of treatment). To maximize clinical relevance as well as clinical homogeneity between studies, our review will be limited to regimens used since 1995, the year in which mycophenolate mofetil (CellCept) and tacrolimus (Prograf) began to be commonly combined in immunosuppression regimens, reflecting a significant change of treatment. This change is associated with significant changes in the approach to immunosuppression since there were corresponding changes in patient characteristics (including age, race and other factors such as human leukocyte antigen (HLA) mismatch status and co-interventions administered), which could have an important impact on the risk of malignancies.

**Table 1:** Summary of agents for consideration in the planned systematic review

**Generic name**	**Trade name**
**Anti-IL2 receptor antagonists**	
Basiliximab	Simulect
Daclizumab	Zenapax
**Polyclonal anti-T-cell antibodies**	
Anti-thymocyte globulin	Thymoglobulin, Atgam
**Monoclonal antibodies**	
Alemtuzumab	Campath, MabCampath, Campath-1H, Lemtrada
Muromonab-CD3	Orthoclone OKT3
**Calcineurin inhibitors**	
Cyclosporin	Neoral, Sandimmune, Restasis, CyA-NOF, CsA-Neoral, CsANeoral
Tacrolimus	Prograf, Advagraf, Protopic, fujimycin, Advagraf, modigraf, LCP-tacro, Tsukubaenolide
**Anti-metabolite agents**	
Azathioprine	Azasan, Imuran, Azamun, Imurel, Azanin, Immuran, Imurek, Muran, Rorasul
Mycophenolate mofetil	CellCept
Mycophenolate sodium	Myfortic
**Mammalian target of rapamycin inhibitors**	
Sirolimus (Rapamycin)	Rapamune
Everolimus	Zortress, Certican
**Co-stimulation blockers**	
Belatacept	Nulojix
**Steroids**	
Prednisone	Adasone, Ancortone, Apo-prednisone, Bicortone, Cartancyl, Colisone, Cortan, Cortancyl, Cortidelt, Cotone, Cutason, Dacorten, Dacortin, Decortancyl, Decortin, Decortisyl, dehydrocortisone, Dekortin, Dellacort, Deltacortene, Deltacortene, Deltacortisone, Delta-cortisone, Deltacortone, Deltasone, Deltra, Di-adreson, DiAdreson, Econosone, Enkorton, Enkortolon, Fernisone, Fiasone, Hostacortin, IN-Sone, Incocortyl, Juvason, Kortancyl, Lisacort, Lodotra, Lodtra, Me-Korti, Metacortandracin, Meticorten, Nisona, Nizon, Novoprednisone, Nurison, Orasone, Panafcort, Panasol, Paracort, Parmenison, Pehacort, Predeltin, Prednicen-M, Prednicorm, Prednicort, Prednicot, Prednidib, Prednilonga, Predniment, predni tablinen, Prednitone, Prednizon, Prednovister, Presone, Pronison, Rayos, Rectodelt, Retrocortine, SK-Prednisone, Servisone, Sone, Sterapred, Supercortil, Winpred, Wojtab, Zenadrid

#### Outcomes

Studies will be considered as eligible if they report on the occurrence of at least one of the following outcomes: PTLD, non-melanoma skin cancer (i.e. squamous cell and basal cell carcinoma) or other cancers (breast, colon, lung, etc.).

#### Study design

Both randomized controlled trials (RCTs) and observational studies will be sought. Reasons for inclusion of both designs are related to both the known limitations of evaluating rare outcomes such as harms based on only RCT data [17-21], and because relevant observational data are available. Recent literature has addressed the value of observational studies to the field of nephrology in addressing several types of issues that RCTs cannot, such as safety issues [22]. We will include observational studies that have enrolled a minimum of 100 patients per treatment group and which (a) use propensity matching techniques to minimize the risk of residual confounding or (b) adjust for a minimum of two of the following key risk factors and report an associated adjusted odds ratio: patient age, smoking status, history of previous cancers, and sun exposure or region of residence (e.g. southern vs northern hemisphere). Studies identifying patients for inclusion based on outcome status will not be eligible. Our objective in selecting observational studies meeting these criteria is to incorporate additional relevant research while seeking those which may be associated with a smaller risk of residual confounding from selection bias.

### Electronic literature search

The literature search strategy has been developed in consultation with a senior information specialist, who received detailed input from the research team. The strategy has been peer reviewed by a second information specialist using the PRESS criteria [23]. A search for RCTs and observational studies was subsequently designed for Medline (1998 to the present), Embase (1998 to the present) and the Cochrane Library. Additional file 1: Table S1 presents the Medline search strategy. Studies will be limited to those published in English, and only published reports will be included.

### Study selection, data collection and risk of bias assessment

Stage 1 screening (i.e. titles and abstracts) will be performed using a liberal accelerated approach, while Stage 2 screening of potentially relevant full text articles will be conducted by two reviewers. Two reviewers will also extract data and assess risk of bias for all studies. Where necessary, a third party will be consulted if disagreements cannot be resolved by discussion amongst the two reviewers with regard to either study selection or data collection. The literature selection process will be presented using a PRISMA flow diagram, and the PRISMA statement will be used to guide the reporting structure [24].

A structured electronic data collection form will be designed and implemented, and it will then be piloted and used by two data collectors. We will collect extensive study-level information including study design; publication characteristics (author, journal, year and so forth); patient inclusion/exclusion criteria; patient demographics of importance to determine the clinical similarity of populations across studies (mean age, gender distribution, prior transplant history, ethnicity, malignancy history, smoking status and the presence of viruses such as the Epstein-Barr virus (EBV), cytomegalovirus, hepatitis B and C viruses, HIV and human T lymphotropic virus); detailed intervention information (agents, doses and duration/frequency of administration, etc.); all clinical outcomes of interest as described previously and risk of bias evaluations. RCTs will be assessed using the Cochrane Risk of Bias tool, and observational studies will be assessed using the Newcastle Ottawa Scale.

### Bias adjustments of observational evidence

The primary bias of interest from observational studies is selection bias, a consequence of how clinicians choose an immunosuppression regimen for their patients, which is based primarily upon a patient’s immunologic risk. For example, higher risk subjects may be given more potent immunosuppression regimens. The risk for organ rejection is determined by the patient’s race, age, sensitization (amount of antibody against anti-human leukocyte antigens) and whether this was the first or a repeat transplantation. The literature suggests that increased age, increased sun exposure and EBV-positive donor organs being transplanted in EBV-negative recipients all increase the risk of malignancy [25-29]. As a result, we may encounter studies where patients in specific intervention groups are more susceptible to malignancies as a result of particular characteristics that are unbalanced between intervention groups (this may also be associated with other unknown confounder imbalances between groups). Methods to assess this adjustment factor are outlined briefly in the analysis section below. Briefly, tables of patient demographic information will be presented to clinical experts in a blinded fashion, who will assess potential selection bias in the included observational studies, which may reflect important differences in the risk of malignancy between intervention groups for each study. This will be done so that the clinicians can judge the lowest and highest perceived differences in the risk of PTLD and other cancers between intervention groups based on differences in baseline characteristics between the patients. Several authors have commented on the need to explore the combination of data across study designs to explore the comparative safety of medical interventions [30-33], and discussions of bias adjustments in meta-analysis have been presented by authors including Eddy *et al*. [34-36], Sutton and Abrams [37], Greenland and Kheifets [38], Turner *et al. *[39], Wolpert and Mengersen [40], Welton and co-workers [41], Dominici *et al. *[42] and others.

### Approach to data analysis

Our approach will be sequential: network meta-analyses will first be performed using RCT data only. The second step will then combine information from randomized and observational studies without bias adjustments. Step 3 will then repeat the analysis from step 2 while incorporating the input of bias adjustments provided by the clinical experts. This approach will allow readers to observe the effect of each step on our findings, and will also allow them to draw their own conclusions based on their preferred level of evidence.

A Bayesian approach will be implemented in WinBUGS (Version 1.4.3, MRC Biostatistics Unit, Cambridge, UK) [43]. Vague prior distributions will be used throughout, allowing the data to drive the final inferences. The outcomes relating to PTLD and cancers will be treated as binary outcomes in primary analyses; however, an approach accounting for variations in person-time and exposure time will be adopted if the duration of follow-up is found to vary substantially from study to study.

Schmitz *et al. *[44] have suggested an approach to combine data from different designs while considering the effect of bias through the down-weighting of observational evidence. We will build on this approach by incorporating our derived study-specific accounts for bias, which will appropriately increase the uncertainty of estimates from observational studies and adjust for any biases identified by expert opinion. To derive these assessments, for each study we will: (i) present blinded demographic tables stratified by intervention group to the evaluating clinical expert; (ii) ask the expert whether, based on his or her own expertise, one intervention group appears to be at greater risk of malignancy than the others based on these demographics, and if yes, why?; (iii) ask the expert what he or she feels is the smallest and the largest percentage difference in the occurrence of malignancy that might be due to the imbalance in patient demographics. These values will populate minimum and maximum values of bias adjustment factors in our sensitivity analyses, which will be drawn from uniform distributions. We will transparently report the experts’ rationale for bias judgments for all studies in our completed review.

All results from network meta-analyses will be reported with point estimates and corresponding 95% credible intervals. The ranking/probability profile of each therapy, the probability of each odds ratio being larger than 1 and the probability of a therapy being associated with the lowest risk of harm will be estimated. Model convergence will be assessed by trace plots and the Brooks-Gelman-Rubin statistic. Three chains will be fit in WinBUGS for each analysis. We will explore model fit and compare alternative models by reviewing the residual deviance and the deviance information criteria from each model. We will look for any statistical inconsistency in the findings from direct and indirect evidence by fitting inconsistency models as described elsewhere [45].

### Supplemental and sensitivity analyses and exploration of heterogeneity

Our sequential approach will allow an assessment of the degree to which estimates vary between randomized and observational studies. We will also use meta-regression and subgroup analyses, if sufficient information is available at the study level. Possible covariates will include year when the study was published, mean patient age, EBV status, race and cytomegalovirus status.

### Reporting findings

We will summarize our estimates using appropriate graphs and tables, along with a layperson’s summary. This will include the following: network diagrams showing the availability of evidence for all possible treatment comparisons; summary point estimates and 95% credible intervals for all pairwise comparisons, both in tabular form and in league tables; and estimates of probabilities that each therapy is deemed ‘best’ for each outcome along with associated average rankings of efficacy and safety [46]. We will also present clear summaries of any deviations from our primary findings that are derived from our planned subgroup and sensitivity analyses.

## Discussion

### What this review will add to the field

The risks of PTLD and other malignancies faced by renal transplant patients as a consequence of the medications needed to minimize the risk of organ rejection are important to both patients and clinicians. We identified no prior systematic review comparing the many possible immunosuppression treatments for kidney transplant patients with regard to the occurrence of these outcomes. Our review will provide a comprehensive source of information in the published literature addressing this issue. From a methodological perspective, there are currently few network meta-analyses combining randomized and observational studies for assessing treatment safety [33]. There is a recent report [44] describing down-weighting methods but it does not appear to address bias at the individual study level. We intend that our review will expand on these methods by incorporating bias adjustments of observational studies assessed at the study level.

### Challenges to the planned review

First, the inclusion of observational studies in meta-analyses has long been debated given concerns of residual confounding in this class of studies. Our review will attempt to address this concern by including only those studies where an effort has been made to reduce this risk, and we will also employ a framework to try to minimize the effect of any remaining confounding in our analyses. Second, the issue of intensity of immunosuppression is an important one that may influence the risk of PTLD and other cancers; the extent to which intensity can be truly established at the patient level within the included studies is unclear at this time and will require further exploration of the included studies to determine. Third, renal transplantation is just one indication for PTLD and cancers resulting from immunosuppression, and it is the indication with the lowest level of risk. Consideration of other indications as well may be worthwhile in the future.

## Abbreviations

EBV: Epstein-Barr virus; HLA: human leukocyte antigen; NMA: network meta-analysis; PICOS: Population – Interventions/Comparators – Outcomes – Study design; PTLD: post-transplant lysmphoproliferative disorder; RCT: randomized controlled trial.

## Competing interests

BH and AT are funded by New Investigator awards from the Canadian Institutes of Health Research and the Drug Safety and Effectiveness Network. BH has provided advice to Amgen Canada regarding methodological issues related to network meta-analysis. The authors declare that they have no competing interests.

## Authors’ contributions

BH conceived the study, selected methods for use, drafted the protocol and gave final approval for the protocol. FY co-drafted the protocol, reviewed drafts of protocol, had final approval of protocol. LJ contributed to the final study design, selected methods for use, reviewed drafts of the protocol and gave final approval for the protocol. JT, MH, MK, NF, CB, AB, DF, DM, AT and SES contributed to the final study design, reviewed drafts of the protocol and gave final approval for the protocol. GK conceived the study, reviewed drafts of the protocol and gave final approval for the protocol.

## Supplementary Material

Additional file 1: Table S1 Literature search strategy. Click here for file
