# Supplementary material for: Checking whether there is an increased risk of post-transplant lymphoproliferative disorder and other cancers with specific modern immunosuppression regimens in renal transplantation: Protocol for a network meta-analysis of randomized and observational studies
Source: Syst Rev. 2014 Feb 22;3:16. doi: 10.1186/2046-4053-3-16 (PMC3936935; doi:10.1186/2046-4053-3-16)
Supplement: Additional file 1: Table S1 — Literature search strategy. [file 2046-4053-3-16-S1.doc]

**Additional file 1: Table S1** Literature Search Strategy

| **Line #** | **Search Criteria** |
| --- | --- |
| 1. | Organ Transplantation/ |
| 2. | (organ? adj3 (graft* or transplant*)).ti,ab. |
| 3. | exp Kidney Transplantation/ |
| 4. | exp Kidney/tr use prmz |
| 5. | ((kidney? or renal) adj3 (allograft? or allotransplant* or graft* or homotransplant* or retransplant* or re-transplant* or transplant*)).ti,ab. |
| 6. | or/1-5 |
| 7. | Immunosuppressive Agents/ use prmz |
| 8. | *Immunosuppressive Agent/ use oemezd |
| 9. | *Immunosuppressive Treatment/ use oemezd |
| 10. | (immunosuppression or immuno-suppression or immune suppression).ti. |
| 11. | (((immunosuppress* or immuno-suppress*) adj (agent? or drug? or substance? or therapy or therapies or treatment?)) or immune suppressant? or immunodepressant? or immuno-depressant? or immunosuppressant? or immuno-suppressant? or immunosuppressor? or immuno-suppressor?).ti,ab. |
| 12. | basiliximab.mp. |
| 13. | 179045-86-4.rn. |
| 14. | (CHI 621 or CHI621 or SDZ-CHI-621 or SDZ-CHI621 or (monoclonal antibody adj (CD25 or CD 25)) or Simulect or Simultec).ti,ab,tn. |
| 15. | abatacept.mp. |
| 16. | 332348-12-6.rn. |
| 17. | (Belatacept or BMS-188667 or BMS188667 or CTLA-4-Ig or Nulojix or CTLA4-Fc or CTLA4-Ig or CTLA4-immunoglobulin or CTLA4Ig or LEA29Y or Orencia or BMS224818 or BMS-224818 or RG2077).ti,ab,tn. |
| 18. | daclizumab.mp. |
| 19. | (dacliximab or dacluzimab or Ro-24-7375 or Zenapax).ti,ab,tn. |
| 20. | Muromonab-CD3/ use prmz |
| 21. | OKT 3/ use oemezd |
| 22. | 140608-64-6.rn. |
| 23. | (CD3-Muromonab or murine monoclonal anti t cell antibody or Muromonab-CD3 or Muromonab or OKT3 or OKT-3 or Orthoclone).ti,ab,tn. |
| 24. | Antilymphocyte Serum/ use prmz |
| 25. | Thymocyte Antibody/ use oemezd |
| 26. | Thymoglobulin*.mp. |
| 27. | ((antilymphoblast? or anti-lymphoblast? or antilymphocyte? or anti-lymphocyte? or antithym* or anti-thym* or antithymocyt* or anti-thymocyt*) adj (antibod* or anti-bod* or antiserum or anti-serum or globulin? or immunoglobulin? or serum?)).ti,ab. |
| 28. | ((lymphocytotoxic adj (antibod* or anti-bod*)) or antilympholin or anti-lympholin or antithymoglobulin? or anti-thymoglobulin?).ti,ab. |
| 29. | (thymocyte adj (antiserum? or anti-serum? or isoantibod* or isoanti-bod* or isoantiserum? or isoanti-serum? or serum?)).ti,ab. |
| 30. | (thymus adj (antibod* or anti-bod* or antiserum? or anti-serum?)).ti,ab. |
| 31. | (Pressimmune or ATGAM or Tecelac).ti,ab,tn. |
| 32. | alemtuzumab.mp. |
| 33. | 216503-57-0.rn. |
| 34. | (Campath* or (CD52 adj monoclonal antibody) or Lemtrada or LDP-103 or LDP103 or MabCambath).ti,ab,tn. |
| 35. | exp Cyclosporins/ use prmz |
| 36. | *Cyclosporine/ use oemezd |
| 37. | Cyclosporin A/ use oemezd |
| 38. | (59865-13-3 or 63798-73-2).rn. |
| 39. | (adi 628 or adi628 or Cicloral or Ciclosporin? or Cipol or Cipol-N or Consupren or Cyclokat or Cyclosporin? or Deximune or Equoral or Gengraf or Implanta or Imusporin or Neoral or Pulminiq or Restasis or Sandimmun? or Sandimun? or sang 35 or sang35 or sangcya or Vekacia or CyA-NOF or CsA-Neoral or CsANeoral or OL 27-400 or OL 27400 or OL27-400 or OL27400).ti,ab,tn. |
| 40. | Tacrolimus/ |
| 41. | (104987-11-3 or 109581-93-3).rn. |
| 42. | (tacrolimus or Advagraf or Avagraf or CCRIS 7124 or Fujimycin or Modigraf or Prograf? or Protopic or Protopy or Tsukubaenolide or FR-900506 or FR900506 or FK-506 or FK506 or L-679934 or LCP-Tacro or UNII-Y5L2157C4J or UNII-WM0HAQ4WNM).ti,ab,tn. |
| 43. | Azathioprine/ |
| 44. | 446-86-6.rn. |
| 45. | (AI3-50290 or Arathioprin? or aza-q or Azafalk or Azahexal or Azamedac or Azamun? or Azanin or Azapin or Azapress or Azaprine or Azarex or Azasan or Azathiodura or Azathiopine or Azathioprim or Azathioprin* or Azathiopurine or Azathropsin or Azatioprin? or Azatox or Azatrilem or Azopi or Azoran or Azothioprin? or Colinsan or Immuran or Immurel or Immuthera or Imunen or Imuprin or Imuran? or Imurel or Imurek or Imuren or Muran or nsc 39084 or nsc39084 or Rorasul or Thioazeprine or Thioprine or Transimune or Zytrim or BW-57-322 or BW-57322 or BW57-322 or BW57322 or CCRIS 62 or Ccucol or EINECS 207-175-4 or HSDB 7084 or NCI-C03474 or NSC-39084 or UNII-MRK240IY2L).ti,ab,tn. |
| 46. | Mycophenolate Mofetil.mp. |
| 47. | Mycophenolic Acid 2 Morpholinoethyl Ester/ use oemezd |
| 48. | (128794-94-5 or 116680-01-4).rn. |
| 49. | (Cellcept or Cell cept or Cellmune or Cellsept or Munoloc or Myclausen or mycophenolic acid mofetil or Myfenax or Myfortic or RS-61443 or RS61443 or HSDB 7436 or UNII-9242ECW6R0).ti,ab,tn. |
| 50. | Mycophenolic Acid/aa use prmz |
| 51. | (mycophenolate adj sodium).mp. |
| 52. | (mycophenolic acid adj (disodium salt or monosodium or sodium)).ti,ab. |
| 53. | ("ERL 080" or ERL080 or ERL 080A or ERL080A or NSC 116072 or UNII-WX877SQI1G).ti,ab. |
| 54. | Prednisone/ |
| 55. | 53-03-2.rn. |
| 56. | (Adasone or Ancortone or Apo-prednisone or Bicortone or Cartancyl or Colisone or Cortan or Cortancyl or Cortidelt or Cotone or Cutason or Dacorten or Dacortin or Decortancyl or Decortin or Decortisyl or dehydrocortisone or Dekortin or Dellacort or Deltacortene or Deltacortene or Deltacortisone or Delta-cortisone or Deltacortone or Deltasone or Deltison? or Deltra or Di-adreson or DiAdreson or Econosone or Encorton? or Enkorton or Enkortolon or Fernisone or Fiasone or Hostacortin or IN-Sone or Incocortyl or Juvason or Kortancyl or Lisacort or Lodotra or Lodtra or Me-Korti or Metacortandracin or Meticorten or Nisona or Nizon or Novoprednisone or Nurison or Orasone or Panafcort or Panasol or Paracort or Parmenison or Pehacort or Predeltin or Prednicen-M or Prednicorm or Prednicort or Prednicot or Prednidib or Prednilonga or Predniment or Prednison* or predni tablinen or Prednitone or Prednizon or Prednovister or Presone or Pronison? or Rayos or Rectodelt or Retrocortine or SK-Prednisone or Servisone or Sone or Sterapred or Supercortil or Ultracorten? or Winpred or Wojtab or Zenadrid).ti,ab,tn. |
| 57. | (AI3-52939 or CCRIS 2646 or EINECS 200-160-3 or HSDB 3168 or NCI-C04897 or NSC 10023 or U 6020 or UNII-VB0R961HZT).ti,ab. |
| 58. | Everolimus.mp. |
| 59. | 159351-69-6.rn. |
| 60. | (Affinitor or Afinitor or Certican or Votubia or Zortress or "RAD 001" or RAD001 or RAD 001a or RAD001a or SDZ-RAD or UNII-9HW64Q8G6G).ti,ab,tn. |
| 61. | Sirolimus/ use prmz |
| 62. | Rapamycin/ use oemezd |
| 63. | 53123-88-9.rn. |
| 64. | (sirolimus or Rapamycin or AY 22-989 or AY 22989 or AY22-989 or AY22989 or CCRIS 9024 or HSDB 7284 or I-2190A or I2190A or NSC 226080 or Rapammune or Rapamune or SILA 9268A or "target-of-rapamycin inhibitors" or UNII-W36ZG6FT64 or WY-090217).ti,ab,tn. |
| 65. | or/7-64 |
| 66. | randomized controlled trial.pt. use prmz |
| 67. | exp Randomized Controlled Trials as Topic/ use prmz |
| 68. | exp Random Allocation/ use prmz |
| 69. | exp Double-Blind Method/ use prmz |
| 70. | exp Single-Blind Method/ use prmz |
| 71. | exp Placebos/ use prmz |
| 72. | Randomized Controlled Trial/ use oemezd |
| 73. | Randomization/ use oemezd |
| 74. | Random Sampling/ use oemezd |
| 75. | Double Blind Procedure/ use oemezd |
| 76. | Single Blind Procedure/ use oemezd |
| 77. | Placebo/ use oemezd |
| 78. | (random* or RCT$1 or placebo*).tw. |
| 79. | ((singl* or doubl* or trebl* or tripl*) adj (mask* or blind* or dumm*)).tw. |
| 80. | Clinical Trial, Phase III.pt. use prmz |
| 81. | Clinical Trial, Phase IV.pt. use prmz |
| 82. | Controlled Clinical Trial.pt. use prmz |
| 83. | Controlled Clinical Trials as Topic/ use prmz |
| 84. | Controlled Clinical Trial/ use oemezd |
| 85. | Phase 3 Clinical Trial/ use oemezd |
| 86. | Phase 4 Clinical Trial/ use oemezd |
| 87. | (control* adj3 trial*).tw. |
| 88. | (non-random* or nonrandom* or quasi-random* or quasirandom*).tw. |
| 89. | ((phase III or phase 3 or phase IV or phase 4) adj3 (study or studies or trial*)).tw. |
| 90. | or/66-89 |
| 91. | Case-Control Studies/ use prmz |
| 92. | Cohort Studies/ use prmz |
| 93. | Cross-Sectional Study/ use prmz |
| 94. | Follow-Up Studies/ use prmz |
| 95. | Longitudinal Studies/ use prmz |
| 96. | Prospective Studies/ use prmz |
| 97. | Retrospective Studies/ use prmz |
| 98. | Registries/ use prmz |
| 99. | exp Case Control Study/ use oemezd |
| 100. | Cohort Analysis/ use oemezd |
| 101. | Controlled Study/ use oemezd |
| 102. | Cross-sectional Study/ use oemezd |
| 103. | Follow Up/ use oemezd |
| 104. | Longitudinal Study/ use oemezd |
| 105. | Observational Study/ use oemezd |
| 106. | Pretest Posttest Control Group Design/ use oemezd |
| 107. | Prospective Study/ use oemezd |
| 108. | Register/ use oemezd |
| 109. | Retrospective Study/ use oemezd |
| 110. | Static Group Comparison/ use oemezd |
| 111. | ((case adj control) or (case adj comparison) or (case adj controlled)).ti,ab. |
| 112. | (case-referent adj3 (study or studies or design or analysis or analyses)).ti,ab. |
| 113. | cohort*.ti,ab. |
| 114. | (control* adj3 (study or studies)).ti,ab. |
| 115. | (cross adj sectional adj7 (study or studies or design or research or analysis or analyses or survey or findings)).ti,ab. |
| 116. | (registries or registry).ti,ab. |
| 117. | ((follow up or followup) adj7 (study or studies or design or analysis or analyses)).ti,ab. |
| 118. | ((follow up or followup) and (base line* or baseline*)).ti,ab. |
| 119. | (longitudinal* adj7 (study or studies or design or analysis or analyses or data)).ti,ab. |
| 120. | (observational* adj3 (study or studies or design or analysis or analyses)).ti,ab. |
| 121. | ((pretest posttest or pre-test posttest or pre-test post-test or pretest post-test) adj3 (study or studies or design)).ti,ab. |
| 122. | (prospective adj7 (study or studies or design or analysis or analyses)).ti,ab. |
| 123. | (retrospective adj7 (study or studies or design or analysis or analyses or data or review)).ti,ab. |
| 124. | (population* adj3 (register? or study or studies or analysis or analyses)).ti,ab. |
| 125. | ((natural adj experiment) or (natural adj experiments)).ti,ab. |
| 126. | ((non experiment or nonexperiment or non experimental or nonexperimental) adj3 (study or studies or design or analysis or analyses)).ti,ab. |
| 127. | ((before-after or (before* adj after)) adj3 (study or studies or design?)).mp. |
| 128. | or/91-127 |
| 129. | exp animals/ |
| 130. | exp animal experimentation/ |
| 131. | exp models animal/ |
| 132. | exp animal experiment/ |
| 133. | nonhuman/ |
| 134. | exp vertebrate/ |
| 135. | or/129-134 |
| 136. | exp humans/ |
| 137. | exp human experimentation/ |
| 138. | exp human experiment/ |
| 139. | or/136-138 |
| 140. | 135 not 139 |
| 141. | Comment.pt. use prmz |
| 142. | Letter.pt. use prmz |
| 143. | Case Reports.pt. use prmz |
| 144. | Letter/ use oemezd |
| 145. | conference abstract.pt. use oemezd |
| 146. | Case Report/ use oemezd |
| 147. | Case Study/ use oemezd |
| 148. | or/141-147 |
| 149. | (6 and 65 and 90) not (140 or 148) |
| 150. | limit 149 to english language |
| 151. | limit 150 to yr="1998 - 2007" |
| 152. | remove duplicates from 151 |
| 153. | limit 150 to yr="2008 -Current" |
| 154. | remove duplicates from 153 |
| 155. | 152 or 154 |
| 156. | (6 and 65 and 128) not (140 or 148) |
| 157. | limit 156 to english language |
| 158. | limit 157 to yr="1998 - 2003" |
| 159. | remove duplicates from 158 |
| 160. | limit 157 to yr="2004 - 2007" |
| 161. | remove duplicates from 160 |
| 162. | limit 157 to yr="2008 - 2011" |
| 163. | remove duplicates from 162 |
| 164. | limit 157 to yr="2012 -Current" |
| 165. | remove duplicates from 164 |
| 166. | 159 or 161 or 163 or 165 |
| 167. | 155 or 166 |
